# Supplementary material for: Olfactory Learning Supports an Adaptive Sugar-Aversion Gustatory Phenotype in the German Cockroach
Source: Insects. 2021 Aug 13;12(8):724. doi: 10.3390/insects12080724 (PMC8397102; doi:10.3390/insects12080724)
Supplement: Supplementary file 1 [file insects-12-00724-s001.zip › insects-1321867-supplementary.pdf]

Table S1. Responses to solvents and innate odor preferences for single compounds

% responding was calculated by the formula: % responding = # of insects making choice / total # of tested insects. % responding among treatments and the numbers of insects attracted to odors from Lure 1 and Lure 2 were tested by Chi-square test (α = 0.05) with Bonferroni corrections. Different letters indicate significant differences Bonferroni corrections (α = 0.05/# of comparisons).

Solvent vs Solvent (% responding)				
Solvent vs Solvent (100 μl/lure)		WT (n)	GA (n)	
Water vs Water		10.0 (20)	10.0 (20)	-
Mineral oil vs Mineral oil		10.0 (20)	10.0 (20)	-
Ethanol vs Ethanol		10.0 (20)	10.0 (20)	-
**Odor vs Solvent (% responding)**				
Odor (μg/solvent/lure) vs Solvent (100 μl/lure)		WT (n)	GA (n)	Chi-square test, Bonferroni
Beta-caryophyllene<br>Solvent: Mineral oil	10^{-4}	20.0 (30) c	33.3 (30) c	$X^2(11) = 99.5$, $p < 0.01$
	10^{-3}	33.3 (30) c	33.3 (30) c	
	0.01	50.0 (30) b	43.3 (30) b	
	0.1	63.3 (30) b	60.0 (30) b	
	1	80.0 (30) a	80.0 (30) a	
	10	100.0 (30) a	100.0 (30) a	
Farnesene<br>Solvent: Mineral oil	10^{-4}	20.0 (30) c	20.0 (30) c	$X^2(11) = 79.6$, $p < 0.01$
	10^{-3}	40.0 (30) b	36.0 (30) b	
	0.01	46.7 (30) b	46.7 (30) b	
	0.1	50.0 (30) b	53.3 (30) b	
	1	66.7 (30) b	66.7 (30) b	
	10	96.7 (30) a	93.3 (30) a	
Benzaldehyde<br>Solvent: Mineral oil	10^{-4}	20.0 (20) b	13.3 (30) b	$X^2(5) = 29.0$, $p < 0.01$
	10^{-3}	36.1 (36) b	26.7 (30) b	
	0.01	50.0 (40) a	33.3 (30) b	
	0.1	45.0 (40) a	33.3 (30) b	
	1	23.0 (61) b	20.0 (30) b	
	10	16.2 (37) b	13.3 (30) b	
3-Methyl-1,2-cyclopentanedione<br>Solvent: Mineral oil	10^{-4}	33.3 (30) c	33.3 (30) c	$X^2(11) = 53.0$, $p < 0.01$
	10^{-3}	50.0 (30) b	43.3 (30) c	
	0.01	56.7 (30) b	56.7 (30) b	
	0.1	60.0 (30) b	53.3 (30) b	
	1	83.3 (30) a	80.0 (30) a	
	10	86.7 (30) a	86.7 (30) a	
4-Hydroxy-3-methoxybenzaldehyde (Vanillin)<br>Solvent: Ethanol	10^{-4}	26.7 (30) c	33.3 (30) c	$X^2(11) = 140.5$, $p < 0.01$
	10^{-3}	56.7 (30) c	56.7 (30) c	
	0.01	70.0 (30) b	66.7 (30) b	
	0.1	100.0 (30) a	100.0 (30) a	
	1	100.0 (30) a	100.0 (30) a	
	10	100.0 (30) a	100.0 (30) a	

4,5-Dimethyl-2-ethylthiazole Solvent: Mineral oil	10^{-8}	30.0 (30)	33.3 (30)	ns
	10^{-7}	36.7 (30)	36.7 (30)	
	10^{-6}	43.3 (30)	46.7 (30)	
	10^{-5}	56.7 (30)	60.0 (30)	
	10^{-4}	53.3 (30)	53.3 (30)	
	10^{-3}	53.3 (30)	53.3 (30)	
2,4,5-Trimethylthiazole Solvent: Mineral oil	10^{-6}	40.0 (30) c	30.0 (30) c	$X^2(11) = 39.8$, $p < 0.01$
	10^{-5}	43.3 (30) b	40.0 (30) b	
	10^{-4}	50.0 (30) b	50.0 (30) b	
	10^{-3}	73.3 (30) b	73.3 (30) b	
	0.01	73.3 (30) b	76.7 (30) a	
	0.1	73.3 (30) b	76.7 (30) a	

Solvent vs Solvent (# of insects making a choice)						
Solvent vs Solvent (100 µl/lure)	WT			GA		
	Left	Right		Left	Right	
Water vs Water	1	1	-	1	1	-
Mineral oil vs Mineral oil	1	1	-	1	1	-
Ethanol vs Ethanol	1	1	-	1	1	-

Odor vs Solvent (# of insects making a choice)							
Odor (µg/solvent/lure) vs Solvent (100 µl/lure)		WT			GA		
		Odor	Solvent	Chi-square test, Bonferroni	Odor	Solvent	Chi-square test, Bonferroni
Beta-caryophyllene Solvent: Mineral oil	10^{-4}	3	3		5	5	
	10^{-3}	5	5		5	5	
	0.01	8	7	ns	7	6	ns
	0.1	12	7		12	6	
	1	17*	7		17	7	
	10	21*	9		22	8	
Farnesene Solvent: Mineral oil	10^{-4}	3	3		3	3	
	10^{-3}	7	5		6	5	
	0.01	8	6	ns	8	6	ns
	0.1	9	6		10	6	
	1	13	7		13	7	
	10	19	10		18	10	
Benzaldehyde Solvent: Mineral oil	10^{-4}	3	3		2	2	
	10^{-3}	6	7		4	4	
	0.01	12	8	ns	6	4	ns
	0.1	11	7		6	4	
	1	7	7		3	3	
	10	3	3		2	2	

3-Methyl-1,2-cyclopentanedione Solvent: Mineral oil	10^{-4}	5	5	ns	5	5	ns
	10^{-3}	8	7		7	6	
	0.01	10	7		10	7	
	0.1	11	7		10	6	
	1	17	8		17*	7	
	10	20*	6		20*	6	
4-Hydroxy-3-methoxybenzaldehyde (Vanillin) Solvent: Ethanol	10^{-4}	4	4	ns	5	5	ns
	10^{-3}	10	7		10	7	
	0.01	14	7		14	6	
	0.1	22*	8		22*	8	
	1	24*	6		24*	6	
	10	24*	6		24*	6	
4,5-Dimethyl-2-ethylthiazole Solvent: Mineral oil	10^{-8}	4	5	ns	4	5	ns
	10^{-7}	6	5		6	5	
	10^{-6}	9	4		9	5	
	10^{-5}	13*	4		14*	4	
	10^{-4}	12*	4		12*	4	
	10^{-3}	12*	4		12*	4	
2,4,5-Trimethylthiazole Solvent: Mineral oil	10^{-6}	5	5	ns	5	5	ns
	10^{-5}	7	6		9	7	
	10^{-4}	9	6		10	5	
	10^{-3}	14	8		14	8	
	0.01	13	9		14	9	
	0.1	12	10		12	11	

Asterisks indicate significant difference between Vanilla and Chocolate in each Treatment (Chi square test, $\alpha = 0.05$).

Table S2. Innate odor preferences for multiple compound blends

% responding was calculated by the formula: % responding = # of insects making choice / total # of tested insects. % responding among treatments and the numbers of insects attracted to odors from Lure 1 and Lure 2 were tested by by Chi-square test (α = 0.05) with Bonferroni corrections. Different letters indicate significant differences Bonferroni corrections (α = 0.05/# of comparisons).

Odor vs Water (% responding)				
Vanilla vs Water		WT (n)	GA (n)	Chi-square test, Bonferroni
Vanilla	0.01 eq[1]	86.7 (30)	86.7 (30)	ns
	0.1 eq	90.0 (30)	90.0 (30)	
	1 eq	86.7 (30)	90.0 (30)	
Chocolate vs Water		WT (n)	GA (n)	Chi-square test, Bonferroni
Chocolate	0.01 eq	83.3 (30)	86.7 (30)	ns
	0.1 eq	90.0 (30)	86.7 (30)	
	1 eq	90.0 (30)	90.0 (30)	

Odor vs Water (# of insects making a choice)							
Vanilla vs Water		WT			GA		
		Odor	Water	Chi-square test, Bonferroni	Odor	Water	Chi-square test, Bonferroni
Vanilla	0.01 eq[1]	19*	7	ns	19*	7	ns
	0.1 eq	23*	4		24*	3	
	1 eq	26*	0		26*	1	
Chocolate vs Water		Odor	Water	Chi-square test, Bonferroni	Odor	Water	Chi-square test, Bonferroni
Chocolate	0.01 eq	20*	5	ns	19*	7	ns
	0.1 eq	23*	4		22*	4	
	1 eq	27*	0		27*	0	

Vanilla vs Chocolate (1 eq[1])				
Cockroach strains	% responding (n)	# of insects that made a choice		Chi-square test Chocolate vs Vanilla
		Chocolate	Vanilla	
WT	90.0 (40)	26*	10	X^2 (1) = 7.1, $p < 0.01$
GA	90.0 (40)	27*	9	X^2 (1) = 9.0, $p < 0.01$
Chi-square test WT vs GA	ns	ns		

[1], Vanilla and Chocolate were used as equivalents of the commercial product extracts, with 1 representing undiluted extracts.

Asterisks indicate significant difference between Vanilla and Chocolate in each Treatment (Chi square test, α = 0.05).

Table S3. Conditioned odor preferences after a single training session to associate an odor with a tastant

% responding was calculated by the formula: % responding = # of insects making choice / total # of tested insects. % responding among treatments and the numbers of insects attracted to odors from Lure 1 and Lure 2 were tested by by Chi-square test (α = 0.05) with Bonferroni corrections. Different letters indicate significant differences Bonferroni corrections (α = 0.05/# of comparisons).

Association of an odor with a tastant (% responding)				
Conditioning (1-session or 3-sessions operant conditioning)		WT (n)	GA (n)	Chi-square test, Bonferroni
Control[1]		90.0 (40)	90.0 (40)	ns
Vanilla + Fructose	1-session	95.0 (40)	90.0 (40)	
	3-sessions	95.0 (40)	92.5 (40)	
Control[1]		90.0 (40)	90.0 (40)	ns
Chocolate + Fructose	1-session	95.0 (40)	92.5 (40)	
	3-sessions	85.0 (40)	95.0 (40)	
Control[1]		90.0 (40)	90.0 (40)	ns
Vanilla + Caffeine	1-session	80.0 (40)	82.5 (40)	
	3-sessions	87.5 (40)	92.5 (40)	
Control[1]		90.0 (40)	90.0 (40)	ns
Chocolate + Caffeine	1-session	82.5 (40)	92.5 (40)	
	3-sessions	85.0 (40)	85.0 (40)	
Control[1]		90.0 (40)	90.0 (40)	ns
Vanilla + Glucose	1-session	90.0 (40)	85.0 (40)	
	3-sessions	85.0 (40)	85.0 (40)	
Control[1]		90.0 (40)	90.0 (40)	ns
Chocolate + Glucose	1-session	90.0 (40)	82.5 (40)	
	3-sessions	87.5 (40)	80.0 (40)	

[1], Control was obtained from Table S2 vanilla and chocolate.

Association of an odor with a tastant (# of insects making a choice)								
Conditioning (1-session or 3-sessions of self training)		WT			GA			
		Chocolate	Vanilla	Chi-square test, Bonferroni	Chocolate	Vanilla	Chi-square test, Bonferroni	
Control[1]		26*	10	a	27*	9	a	
Vanilla + Fructose	1-session	15	23	b	16	20	b	
	3-sessions	12	26*	c	17	20	b	
		$X^2(2) = 13.7, p < 0.01$			$X^2(2) = 8.7, p = 0.01$			
Control[1]		26*	10	a	27*	9	a	
Chocolate + Fructose	1-session	35*	3	b	34*	3	b	
	3-sessions	32*	2	b	36*	2	b	
		$X^2(2) = 8.8, p = 0.01$			$X^2(2) = 7.6, p = 0.02$			
Control[1]		26*	10	a	27*	9	a	
Vanilla + Caffeine	1-session	24*	8	a	26*	7	a	
	3-sessions	33*	2	b	36*	1	b	
		$X^2(2) = 6.4, p = 0.03$			$X^2(2) = 7.7, p = 0.02$			
Control[1]		26*	10	a	27*	9	a	
Chocolate + Caffeine	1-session	25*	8	a	25	12	b	
	3-sessions	13	21	b	14	20	c	
		$X^2(2) = 12.4, p < 0.01$			$X^2(2) = 9.3, p < 0.01$			
Control[1]		26*	10	a	27*	9	a	
Vanilla + Glucose	1-session	16	20	b	27*	7	a	
	3-sessions	15	19	b	33*	1	b	
		$X^2(2) = 7.5, p = 0.02$			$X^2(2) = 6.9, p < 0.05$			
Control[1]		26*	10	a	27*	9	a	
Chocolate + Glucose	1-session	34*	2	b	24*	9	a	
	3-sessions	33*	2	b	13	19	b	
		$X^2(2) = 10.3, p < 0.01$			$X^2(2) = 10.5, p < 0.01$			

[1], Control was obtained from Table S2 vanilla and chocolate.

Asterisks indicate significant difference between Vanilla and Chocolate in each Treatment (Chi square test, $\alpha = 0.05$).

Table S4. Conditioned odor preferences after training to associate two odors with different tastants

% responding was calculated by the formula: % responding = # of insects making choice / total # of tested insects. % responding among treatments and the numbers of insects attracted to odors from Lure 1 and Lure 2 were tested by by Chi-square test (α = 0.05) with Bonferroni corrections. Different letters indicate significant differences Bonferroni corrections (α = 0.05/# of comparisons).

Association of two odors with different tastants (% responding)				
Conditioning (1-session or 3-sessions of self training)		WT (n)	GA (n)	Chi-square test, Bonferroni
Control[1]		90.0 (40)	90.0 (40)	ns
Vanilla + Fructose	1-session	93.8 (80)	84.0 (50)	
Chocolate + Caffeine	3-sessions	90.0 (50)	87.5 (40)	
Control[1]		90.0 (40)	90.0 (40)	ns
Vanilla + Caffeine	1-session	93.3 (105)	94.0 (50)	
Chocolate + Fructose	3-sessions	81.3 (80)	96.0 (50)	
Control[1]		90.0 (40)	90.0 (40)	ns
Vanilla + Fructose	1-session	88.7 (62)	87.5 (40)	
Chocolate + Glucose	3-sessions	84.0 (50)	88.6 (70)	
Control[1]		90.0 (40)	90.0 (40)	ns
Vanilla + Glucose	1-session	85.0 (60)	96.4 (55)	
Chocolate + Fructose	3-sessions	85.0 (60)	92.3 (65)	
Control[1]		90.0 (40)a	90.0 (40)a	$X^2(5) = 64.1, p < 0.01$
Vanilla + Glucose	1-session	83.3 (60)a	57.1 (70)b	
Chocolate + Caffeine	3-sessions	87.7 (57)a	42.5 (80)b	
Control[1]		90.0 (40)a	90.0 (40)a	$X^2(1) = 163.7, p < 0.01$
Vanilla + Caffeine	1-session	92.9 (70)a	65.7 (70)b	
Chocolate + Glucose	3-sessions	92.5 (80)a	37.1 (70)b	

[1], Control was obtained from Table S2 vanilla and chocolate.

Association of two odors with different tastants (# of insects making a choice)							
Conditioning (1-session or 3-sessions of self training)		WT			GA		
		Chocolate	Vanilla	Chi-square test, Bonferroni	Chocolate	Vanilla	Chi-square test, Bonferroni
Control[1]		26*	10	a	27*	9	a
Vanilla + Fructose Chocolate + Caffeine	1-session	35	40	b	21	21	b
	3-sessions	8	37*	c	8	27*	c
		$X^2(2) = 24.4, p < 0.01$			$X^2(2) = 19.3, p < 0.01$		
Control[1]		26*	10	a	27*	9	a
Vanilla + Caffeine Chocolate + Fructose	1-session	82*	16	b	42*	5	b
	3-sessions	60*	5	c	45*	3	b
		$X^2(2) = 7.2, p = 0.03$			$X^2(2) = 6.8, p = 0.03$		
Control[1]		26*	10	-	27*	9	a
Vanilla + Fructose Chocolate + Glucose	1-session	31	24	-	18	17	b
	3-sessions	22	20	-	15	47*	c
		ns			$X^2(2) =24.5, p < 0.01$		
Control[1]		26*	10	-	27*	9	a
Vanilla + Glucose Chocolate + Fructose	1-session	25	26	-	47*	6	b
	3-sessions	25	26	-	58*	2	c
		ns			$X^2(2) = 10.5, p < 0.01$		
Control[1]		26*	10	a	27*	9	-
Vanilla + Glucose Chocolate + Caffeine	1-session	23	27	b	29*	11	-
	3-sessions	11	39*	c	25*	9	-
		$X^2(2) = 21.5, p < 0.01$			ns		
Control[1]		26*	10	a	27*	9	-
Vanilla + Caffeine Chocolate + Glucose	1-session	57*	8	b	34*	12	-
	3-sessions	69*	5	c	19*	7	-
		$X^2(2) = 9.4, p < 0.01$			ns		

[1], Control was obtained from Table S2 vanilla and chocolate.

Asterisks indicate significant difference between Vanilla and Chocolate in each Treatment (Chi square test, α = 0.05).

Table S5. Retention of olfactory memory

% responding was calculated by the formula: % responding = # of insects making choice / total # of tested insects. % responding among treatments and the numbers of insects attracted to odors from Lure 1 and Lure 2 were tested by by Chi-square test (α = 0.05) with Bonferroni corrections. Different letters indicate significant differences Bonferroni corrections (α = 0.05/# of comparisons).

Retention of olfactory memory (% responding)				
Conditioning (3 sessions, self training)	Tested day	WT (n)	GA (n)	Chi-square test, Bonferroni
Control[1]		90.0 (40)	90.0 (40)	ns
Vanilla + Fructose Chocolate + Caffeine	1-d later[2]	90.0 (50)	87.5 (40)	
	2-d later	92.5 (40)	92.5 (40)	
	3-d later	92.5 (40)	92.5 (40)	
	5-d later	92.5 (40)	92.5 (40)	
Control[1]		90.0 (40)	90.0 (40)	ns
Vanilla + Fructose Chocolate + Glucose	1-d later[2]	84.0 (50)	88.6 (70)	
	2-d later	95.0 (40)	97.5 (40)	
	3-d later	95.0 (40)	97.5 (40)	
	5-d later	95.0 (40)	95.0 (40)	
Control[1]		90.0 (40) a	90.0 (40) a	$X^2(9) = 113.4, p < 0.01$
Vanilla + Glucose Chocolate + Caffeine	1-d later[2]	87.7 (57) a	42.5 (80) b	
	2-d later	90.0 (40) a	42.5 (40) b	
	3-d later	92.5 (40) a	70.0 (40) c	
	5-d later	97.5 (40) a	90.0 (40) a	

[1], Control was obtained from Table S2 vanilla and chocolate.
[2], Conditioned preference 1-d later was obtained from Table S4.

Retention of olfactory memory (# of insects making a choice)							
Conditioning (3 sessions, self training)	Tested day	WT			GA		
		Chocolate	Vanilla	Chi-square test, Bonferroni	Chocolate	Vanilla	Chi-square test, Bonferroni
Control[1]		26*	10	a	27*	9	a
Vanilla + Fructose and Chocolate + Caffeine	1-d later[2]	8	37*	b	8	27*	b
	2-d later	11	26*	b	10	27*	b
	3-d later	15	22	c	16	21	c
	5-d later	25*	12	a	26*	11	a
		$X^2(4) = 35.7$, $p < 0.01$			$X^2(4) = 33.6$, $p < 0.01$		
Control[1]		26*	10	-	27*	9	a
Vanilla + Fructose and Chocolate + Glucose	1-d later[2]	22	20	-	15	47*	b
	2-d later	20	18	-	13	26*	b
	3-d later	20	18	-	20	19	c
	5-d later	22	16	-	27*	11	a
		ns			$X^2(4) = 36.2$, $p < 0.01$		
Control[1]		26*	10	a	27*	9	-
Vanilla + Glucose and Chocolate + Caffeine	1-d later[2]	11	39*	b	25*	9	-
	2-d later	13	23*	c	12	5	-
	3-d later	18	19	c	21*	7	-
	5-d later	24	15	a	26*	10	-
		$X^2(4) = 26.8$, $p < 0.01$			ns		

[1], Control was obtained from Table S2 vanilla and chocolate.

[2], Conditioned preference 1-d later was obtained from Table S4.

Asterisks indicate significant difference between Vanilla and Chocolate in each Treatment (Chi square test, $\alpha = 0.05$).
